# Hepatocyte Sirtuin 6 Protects against Atherosclerosis and Steatohepatitis by Regulating Lipid Homeostasis

**DOI:** 10.3390/cells12152009

**Published:** 2023-08-05

**Authors:** Yingdong Zhu, Shuwei Hu, Xiaoli Pan, Raja Gopoju, Fathima N. Cassim Bawa, Liya Yin, Yanyong Xu, Yanqiao Zhang

**Affiliations:** 1 Department of Integrative Medical Sciences, Northeast Ohio Medical University, Rootstown, OH 44272, USArgopoju@neomed.edu (R.G.);; 2 School of Biomedical Sciences, Kent State University, Kent, OH 44240, USA

**Keywords:** Sirtuin 6, NAFLD, atherosclerosis, obesity, bile acid, lipid metabolism

## Abstract

Histone deacetylase Sirtuin 6 (SIRT6) regulates many biological processes. SIRT6 is known to regulate hepatic lipid metabolism and inhibit the development of nonalcoholic fatty liver disease (NAFLD). We aimed to investigate the role of hepatocyte SIRT6 in the development of atherosclerosis and further characterize the mechanism underlying SIRT6’s effect on NAFLD. *Ldlr*^−/−^ mice overexpressing or lacking hepatocyte SIRT6 were fed a Western diet for 16 weeks. The role of hepatic SIRT6 in the development of nonalcoholic steatohepatitis (NASH), atherosclerosis, and obesity was investigated. We also investigated whether p53 participates in the pathogenesis of NAFLD in mice overexpressing hepatic SIRT6. Our data show that loss of hepatocyte SIRT6 aggravated the development of NAFLD, atherosclerosis, and obesity in *Ldlr*^−/−^ mice, whereas adeno-associated virus (AAV)-mediated overexpression of human SIRT6 in the liver had opposite effects. Mechanistically, hepatocyte SIRT6 likely inhibited the development of NAFLD by inhibiting lipogenesis, lipid droplet formation, and p53 signaling. Hepatocyte SIRT6 also likely inhibited the development of atherosclerosis by inhibiting intestinal lipid absorption and hepatic VLDL secretion. Hepatic SIRT6 also increased energy expenditure. In conclusion, our data indicate that hepatocyte SIRT6 protects against atherosclerosis, NAFLD, and obesity by regulating lipid metabolism in the liver and intestine.

## 1. Introduction

Dysregulation of the epigenome drives aberrant transcriptional programs that promote disease onset and progression. Histone modification is a covalent post-translational modification that includes acetylation, methylation, phosphorylation, ubiquitylation, and sumoylation. Histone acetylation is regulated by adding or removing acetyl-CoA via histone acetyltransferases (HATs) and histone deacetylases (HDACs) in the lysine residues, respectively. Sirtuin 6 (SIRT6) is one of the NAD^+^-dependent sirtuins (SIRT1-7) that belong to class III HDACs. SIRT6, a stress-responsive protein deacetylase of both acetyl groups and long-chain fatty-acyl groups, is also a mono-ADP-ribosyltransferase that transfers ADP–ribose moieties to the lysine and arginine residues of protein substrates [1]. SIRT6 regulates cellular homeostasis by modulating DNA repair, telomere maintenance, and lipid and glucose metabolism. Therefore, it participates in a plethora of diseases such as aging, cancer, fatty liver disease, cardiovascular disease, obesity, diabetes, neurodegeneration, etc. [1,2]. 

The role of hepatic SIRT6 in lipid metabolism has been extensively investigated. Kim et al. showed that mice lacking hepatic *Sirt6* accumulate hepatic triglyceride (TG) due to inhibition of genes involved in lipogenesis and glycolysis and induction of genes involved in fatty acid oxidation (FAO) [3]. Naiman et al. showed that SIRT6 promotes hepatic FAO via PPARα [4]. Recently, Zhu et al. showed that mice lacking hepatic *Sirt6* develop severe fatty livers when fed a Western diet and that Sirt6 inhibits lipogenesis by suppressing liver X receptor (LXR), carbohydrate response element-binding protein (ChREBP), and sterol regulatory element-binding protein 1 (SREBP1) [5]. Tao et al. reported that SIRT6 reduces LDL-C levels by inhibiting proprotein convertase subtilisin/kexin type 9 (PCSK9)-mediated LDL receptor degradation [6] and that SIRT6 lowers hepatic cholesterol levels by repressing SREBP2 [7]. 

In addition to regulating lipid metabolism, SIRT6 inhibits liver fibrogenesis via diverse mechanisms. In hepatic stellate cells (HSC), SIRT6 inhibits HSC activation via the deacetylation of SMAD family member 2 (Smad2) [8], Smad3 [9], transforming growth factor β (TGFβ) [10], Yes-associated protein (YAP), and transcriptional coactivator with PDZ-binding motif (TAZ) [11]. Ka et al. showed that SIRT6 partially protects against high fat/high fructose-induced nonalcoholic steatohepatitis (NASH) by regulating nuclear factor erythroid 2-related factor 2 (NRF2)-mediated attenuation of oxidative stress [12]. 

Despite significant research conducted on the role of hepatic SIRT6 in metabolic regulation, the role of hepatic SIRT6 in atherosclerosis or obesity has not been explored. In addition, the role of hepatic SIRT6 in NASH development is not fully understood. In this study, we used mice over-expressing or lacking hepatic SIRT6 to show that hepatic SIRT6 protects against Western diet-induced atherosclerosis, steatohepatitis, and obesity. The atheroprotective effect of hepatic SIRT6 is independent of LDLR. Furthermore, we reveal novel mechanisms contributing to hepatic SIRT6-mediated NASH development and atherogenesis. 

## 2. Materials and Methods

### 2.1. Mice and Diets

C57BL/6J mice, albumin-cre (alb-cre) mice (stock # 003574), and *Ldlr*^−/−^ mice (stock # 002207) were purchased from Jackson Laboratory (Bar Harbor, ME, USA) on a C57BL/6J background. The *Sirt6^fl/fl^* mice were described previously [3]. *Sirt6^fl/fl^* mice were crossed with albumin-Cre mice to generate liver-specific *Sirt6*^−/−^ mice (*Sirt6^Hep^*^−/−^) and control (*Sirt6^fl/fl^*) mice. *Sirt6^fl/fl^* mice and *Ldlr*^−/−^ mice were cross-bred to generate *Sirt6^fl/fl^Ldlr*^−/−^ mice. A Western diet containing 21% fat/0.2% cholesterol (stock # TD.88137) was purchased from Envigo (Indianapolis, IN, USA). Unless otherwise stated, about two-month-old male mice were fed this special diet for four months and fasted for 5–6 h during the light cycle prior to anesthesia. All animal studies complied with the ARRIVE guidelines and were approved by the Institutional Animal Care and Use Committee at Northeast Ohio Medical University. 

### 2.2. Adeno-Associated Virus

The coding sequence of human SIRT6 was amplified by high-fidelity PCR and cloned into an AAV vector under the control of a mouse albumin promoter (AAV-ALB-hSirt6). Vector Biolabs (Malvern, PA, USA) produced and titrated AAV8-ALB-Null (control), AAV8-ALB-hSIRT6, AAV8-TBG-Null, and AAV8-TBG-Cre. Each mouse was injected intravenously with 2 × 10^11^ genome copies of AAVs.

### 2.3. Real-Time PCR

Total RNA was isolated from the liver using Trizol (Invitrogen; cat #15596018). The genomic DNA was removed using the DNA-free™ Kit (Ambion; cat # AM1906). The cDNA was generated following the instructions of the TaqMan Reverse Transcription Kit (Applied Biosystems; Waltham, MA, USA; cat# N8080234). qPCR was performed using the PowerUp SYBR Green master mix (ThermoFisher Scientific; Waltham, MA, USA: cat# A25778) on a 7500 real-time PCR machine (Applied Biosystems; Waltham, MA, USA). Relative mRNA levels were quantified using the 2^−ΔΔCt^ method normalized to *36b4*. 

### 2.4. Western Blot

Western blot assays were performed using whole liver lysates and nuclear or microsomal protein lysates from the liver samples. Antibodies against mouse (# NB100-2522) or human (# D8012) SIRT6 were purchased from Novus Biologicals (Centennial, CO, USA) and Cell Signaling (Danvers, MA, USA), respectively. Antibodies against mouse P53 (# ab26 and # ab131442), human P53 (# ab246550), and Tubulin (# ab4074) were purchased from Abcam (Cambridge, Cambridgeshire, UK). Antibodies against human P53 (# SC-126) or Histone (# SC-517576) were purchased from Santa Cruz (Dallas, TX, USA). Antibodies against CYP7A1 (# TA351400) or CYP8B1 (# TA313734) were purchased from Origene (Rockville, MD, USA). The antibody against calnexin (#NB100-1965) was purchased from Novus (Centennial, CO, USA). Peroxidase-conjugated secondary antibodies were from Jackson ImmunoResearch Laboratories (West Grove, PA, USA). Primary antibodies were diluted at 1:1000 except for the Tubulin antibody, which was diluted at 1:5000. 

### 2.5. Liver Histology and Apoptosis Assays

Fresh liver samples were fixed in 10% formalin. Livers were dehydrated and frozen-sectioned by Cryostat (Leica CM1950; Deer Park, IL, USA) for Oil red O staining. Livers were paraffin-embedded and sectioned by microtome (Leica RM2235) for hematoxylin and eosin (H&E) staining, picrosirius red staining, or TUNEL assay. Staining of apoptotic nuclei was performed using a TUNEL assay kit (ab206386; Abcam; Cambridge, Cambridgeshire, UK). 

### 2.6. Hepatic Lipids and Hydroxyproline

Hepatic total lipids were extracted from chloroform/methanol (2:1 *v*/*v*) using the Bligh and Dyer method, as described previously [13]. We then assessed triglyceride concentrations from the resulting emulsion using Infinity reagents (Thermo Fisher Scientific; Waltham, MA, USA). Free cholesterol and free fatty acid concentrations were measured according to the manufacturer’s instructions (Fujifilm; Tokyo, Japan). We weighed a portion of fresh or frozen liver and used a kit from Cell Biolabs (STA675) to measure hepatic hydroxyproline levels. The result was expressed as μg/mg liver. 

### 2.7. Cell Culture and Transfection

Plasmids were transfected into Hepa1-6 cells using Lipofectamine 3000 transfection kit (Invitrogen; Carlsbad, CA, USA; cat # L3000015) or FuGene HD transfection reagent (Promega; Madison, WI, USA; cat # E2311). The cells were cultured in Dulbecco’s Modified Eagle Medium (DMEM) containing 10% fetal bovine serum (FBS), 1 mM sodium pyruvate, and 1× antibiotic-antimycotic (Gibco; Carlsbad, CA, USA).

### 2.8. Plasma Lipid, ALT, AST, and Lipoprotein Profile Assays

Plasma cholesterol (cat # TR13421), triglyceride (cat # TR22421), alanine aminotransferase (ALT; cat # TR71121), and aspartate aminotransferase (AST; cat # TR70121) levels were measured using Infinity reagents (ThermoFisher Scientific; Waltham, MA, USA). Plasma lipoproteins were separated by fast protein liquid chromatography (FPLC). In brief, more than 100 μL plasma was run at 0.5 mL/min in a buffer (0.15 mol/L NaCl, 0.01 mol/L Na2HPO4, 0.1 mmol/L EDTA, pH 7.5), and lipoproteins were separated on a Superose 6 10/300 GL column (GE Healthcare; Chicago, IL, USA) using BioLogic DuoFlow QuadTec 10 System (Bio-Rad; Hercules, CA, USA). The total cholesterol or triglyceride amount in each fraction (500 μL) was calculated after a small portion (50 μL) was used for quantification. 

### 2.9. VLDL Secretion

Mice were fasted for 5 h, followed by an intravenous injection of tyloxapol (500 mg/kg). Blood was collected at various time points (0, 30, 60, 90, 120, and 180 min) and triglyceride levels were quantified. 

### 2.10. Intestinal Fat Absorption

Mice were fasted for at least 4 h, followed by an intravenous injection of tyloxapol (500 mg/kg). The mice were then gavaged with olive oil (15 μL/g body weight). Blood samples were drawn at various time points and triglyceride levels were quantified as described [14,15].

### 2.11. Intestinal Cholesterol Absorption

Mice were i.v. injected with 2.5 μCi ^3^H-cholesterol in Intralipid (Sigma; St. Louis, MO, USA), followed by immediate gavaging with 1 μCi ^14^C-cholesterol in median-chain triglycerides (MCT oil; Mead Johnson, Evansville, IN, USA). After 72 h, blood and tissue were collected. Plasma was collected to determine ^3^H and ^14^C activity. Cholesterol absorption was calculated as previously described [14,16].

### 2.12. Bile Acid Measurement

The total bile acids in the liver, intestine, and gallbladder were extracted in ethanol as described [17]. The bile acid concentration was quantified using the total bile acid assay kit from Diazyme (Poway, CA, USA; cat # DZ042AK01). We calculated the bile acid pool size based on the total amount of bile acids in the liver, intestine, and gallbladder. 

### 2.13. Atherosclerotic Lesion Quantification

The whole aorta, including the ascending, thoracic, and abdominal segments, was isolated and cleaned under a microscope. The en face aortas and sectioned aortic roots were stained with Oil red O. The atherosclerotic plaque size was determined using ImageJ software from National Institutes of Health (Bethesda, MD, USA). The lesion was selected by the “Freehand tool”, and the lesion areas in μm^2^ were collected using “Control + M”. 

### 2.14. Body Composition and Energy Expenditure

We used EchoMRI™-700 (EchoMRI LLC, Houston, TX, USA) to measure the whole body fat and lean masses of the mice. The Comprehensive Lab Animal Monitor System (CLAMS) system was used to measure oxygen consumption and heat production, as described previously [18]. In brief, mice underwent an acclimation period, and 24 h measurement of energy expenditure was determined using an eight-chamber system. Each run included two genotypes with four mice per group. 

### 2.15. Statistical Analysis

Statistical significance was analyzed using a student *t*-test or two-way ANOVA by Prism (GraphPad, Boston, MA, USA). All values were expressed as mean ± SEM. Differences were considered statistically significant at *p* < 0.05.

## 3. Results

### 3.1. Hepatocyte SIRT6 Is Required for Protection against Western Diet-Induced Steatohepatitis

**Figure 1:**
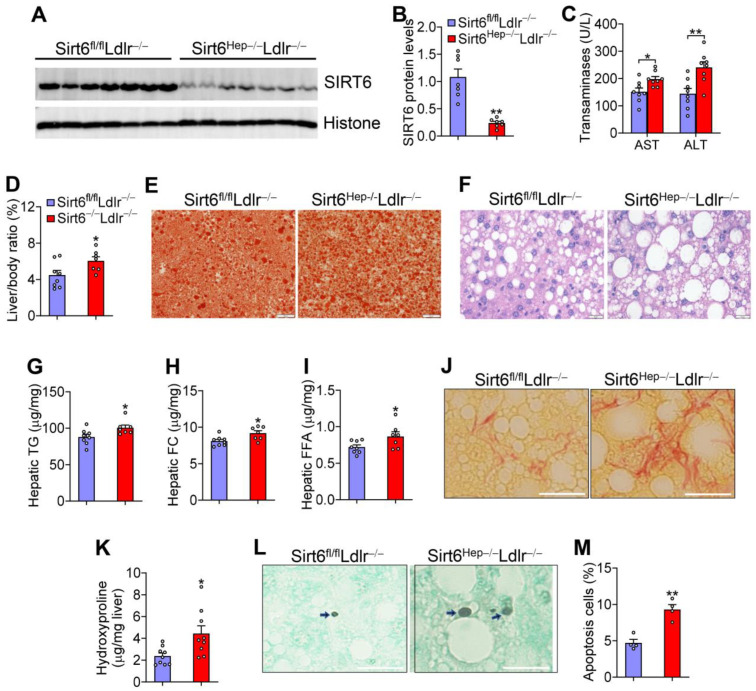
**Loss of hepatic Sirt6 in *Ldlr*^−/−^ mice aggravates Western diet-induced steatohepatitis.** *Sirt6^fl/fl^Ldlr*^−/−^ mice and *Sirt6^Hep^*^−/−^*Ldlr*^−/−^ mice were fed a Western diet for 16 weeks (n = 8 per group). (**A**,**B**) Western blot assays were performed (**A**) and SIRT6 protein levels were quantified (**B**). (**C**) Plasma AST and ALT levels. (**D**) The ratio of liver to body weight (%). (**E**,**F**) Oil red O (**E**) and H&E (**F**) staining of liver sections. (**G**–**I**) Hepatic triglyceride (TG) (**G**), free cholesterol (FC) (**H**), and free fatty acid (FFA) (**I**) levels. (**J**) Picrosirius red staining of liver sections. (**K**) Hepatic hydroxyproline levels. (**L**) TUNEL staining. Arrows point to staining-positive cells. (**M**) Percentage of apoptotic cells. Scale bars: 20 μm in (**E**,**F**,**J**,**L**). H&E, Sirius red, TUNEL. All data are expressed as mean ± SEM. Data points in the graphs represent an individual mouse or a biological measurement. Statistical analysis was performed using a student *t*-test. * *p* < 0.05, ** *p* < 0.01.

The role of hepatic SIRT6 in the development of diet-induced steatohepatitis has not been fully clarified to date. Hyperlipidemic *Ldlr*^−/−^ mice develop severe liver steatosis, inflammation, obesity, and insulin resistance when fed a Western diet. For this reason, they have been used to study the development of NASH [19] and atherosclerosis. Therefore, we crossed *Sirt6^fl/fl^* mice with *Ldlr*^−/−^ mice to generate *Sirt6^fl/fl^Ldlr*^−/−^ mice, which were then i.v. injected with AAV8-TBG-Cre or AAV8-TBG-Null to generate *Ldlr*^−/−^ mice with a hepatocyte-specific deletion of *Sirt6* (*Sirt6^Hep^*^−/−^*Ldlr*^−/−^) and the control (*Sirt6^fl/fl^Ldlr*^−/−^) mice, respectively. These mice were fed a Western diet for 16 weeks. Compared to *Sirt6^fl/fl^Ldlr*^−/−^ mice, *Sirt6^Hep^*^−/−^*Ldlr*^−/−^ mice had a 77% reduction in hepatic SIRT6 protein levels (Figure 1A,B). *Sirt6^Hep^*^−/−^*Ldlr*^−/−^ mice had increased plasma AST and ALT levels (Figure 1C), and the ratio of liver to body weight (Figure 1D). Oil red O staining (Figure 1E) and H & E staining (Figure 1F) showed that *Sirt6^Hep^*^−/−^*Ldlr*^−/−^ mice had increased lipid accumulation. Further biochemical quantification data showed that hepatic triglyceride (TG) (Figure 1G), free cholesterol (FC) (Figure 1H), and free fatty acid (FFA) (Figure 1I) levels were increased in *Sirt6^Hep^*^−/−^*Ldlr*^−/−^ mice. *Sirt6^Hep^*^−/−^*Ldlr*^−/−^ mice also had increased fibrosis (Figure 1J) and hepatic hydroxyproline levels (Figure 1K). Finally, *Sirt6^Hep^*^−/−^*Ldlr*^−/−^ mice had increased hepatic apoptosis (Figure 1L,M). Thus, the data in Figure 1 demonstrate that hepatocyte SIRT6 is required for protection against diet-induced steatohepatitis.

### 3.2. Hepatic Expression of Human SIRT6 Prevents Western Diet-Induced Steatohepatitis

**Figure 2:**
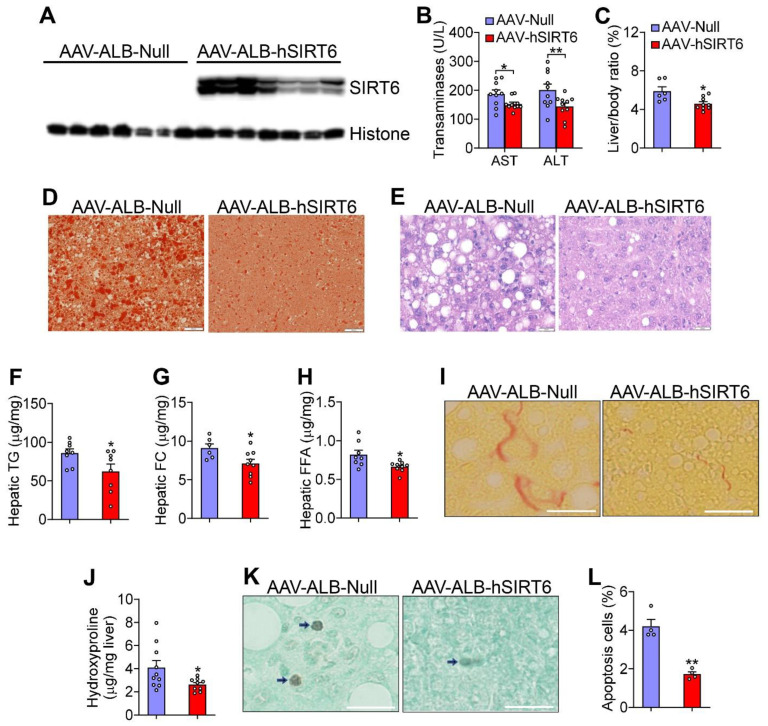
**Over-expression of hepatic SIRT6 in *Ldlr*^−/−^ mice protects against Western diet-induced steatohepatitis.** *Ldlr*^−/−^ mice were i.v. injected with AAV8-ALB-Null or AAV8-ALB-hSIRT6 and then fed a Western diet for 16 weeks (n = 8 per group). (**A**) Western blot assays were performed. (**B**) Plasma AST and ALT levels. (**C**) The ratio of liver to body weight (%). (**D**,**E**) Oil red O (**D**) and H&E (**E**) staining of liver sections. (**F**–**H**) Hepatic triglyceride (TG) (**F**), free cholesterol (FC) (**G**), and free fatty acid (FFA) (**H**) levels. (**I**) Picrosirius red staining of liver sections. (**J**) Hepatic hydroxyproline levels. (**K**) TUNEL staining. Arrows point to stain-positive cells. (**L**) Percentage of apoptotic cells. Scale bars: 20 μm in (**D**,**E**,**I**,**K**). All data are expressed as mean ± SEM. Data points in the graphs represent an individual mouse or a biological measurement. Statistical analysis was performed using a student *t*-test. * *p* < 0.05, ** *p* < 0.01.

To address whether hepatic overexpression of SIRT6 regulates the development of NAFLD, we generated an AAV expressing human SIRT6 under the control of an albumin promoter (AAV8-ALB-hSIRT6). When fed a Western diet for 16 weeks, the hepatic expression of human SIRT6 in *Ldlr*^−/−^ mice (Figure 2A) reduced plasma AST and ALT levels (Figure 2B) and the ratio of liver to body weight (Figure 2C). Hepatic overexpression of human SIRT6 also reduced hepatic neutral lipid accumulation (Figure 2D,E), TG, FC, and FFA levels (Figure 2F–H), fibrosis (Figure 2I,J), and apoptosis (Figure 2K,L). Thus, the data in Figure 1 and Figure 2 demonstrate that hepatocyte SIRT6 protects against Western diet-induced steatohepatitis in *Ldlr*^−/−^ mice. 

### 3.3. Hepatic SIRT6 Inhibits Genes Involved in Lipogenesis, Lipid Droplet Formation, Inflammation, and Fibrogenesis

**Figure 3:**
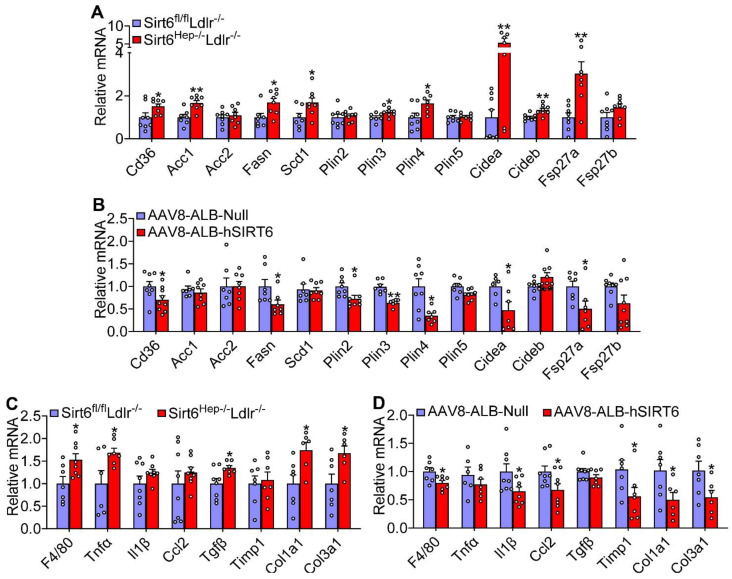
**Hepatic SIRT6 inhibits genes involved in lipogenesis, lipid droplet formation, inflammation, and fibrogenesis.** Hepatic mRNA levels in *Ldlr*^−/−^ mice lacking (**A**,**C**) or over-expressing (**B**,**D**) hepatic SIRT6 were determined (n = 8 per group). All data are expressed as mean ± SEM. Data points in the graphs represent an individual mouse or a biological measurement. Statistical analysis was performed using a student *t*-test. * *p* < 0.05, ** *p* < 0.01.

To investigate the mechanisms underlying steatohepatitis regulation by SIRT6, we analyzed the hepatic expression of genes related to the development of NAFLD. In *Ldlr*^−/−^ mice lacking hepatocyte *Sirt6*, a number of genes were induced, including genes involved in fatty acid uptake (cluster of differentiation 36 (*Cd36*)), lipogenesis (acetyl-CoA carboxylase 1 (*Acc1*), fatty acid synthase (*Fasn*), stearoyl-CoA desaturase 1 (*Scd1*), and lipid droplet formation (perilipin 3 (*Plin3*), *Plin4*, cell death inducing DFFA like effector a (*Cidea*), *Cideb*, and fat-specific Protein 27a (*Fsp27a*)) (Figure 3A). By contrast, over-expression of human SIRT6 in *Ldlr*^−/−^ mice had opposite effects on these genes (Figure 3B). In addition, the inactivation of hepatocyte *Sirt6* induced hepatic F4/80, tumor necrosis factor-alpha (*Tnfα*), transforming growth factor beta (*Tgfβ*), collagen 1a1 (*Col1a1*), and *Col3a1* (Figure 3C), whereas these changes were largely reversed in mice overexpressing hepatic SIRT6 (Figure 3D). These data suggest that hepatic SIRT6 likely inhibits the development of NAFLD by inhibiting lipogenesis, lipid droplet formation, inflammation, and fibrogenesis. 

### 3.4. SIRT6 Reduces Hepatic Apoptosis and Lipid Levels Partly via p53

Apoptosis is believed to play an important role in NASH development by triggering hepatic inflammation [20]. The tumor suppressor protein p53 is involved in DNA repair and apoptosis [21] and the pathogenesis of NAFLD [22,23]. SIRT6 is shown to deacetylate lysine 382 of p53 [24]. Interestingly, overexpression of SIRT6 reduced p53 expression by >71% in Hepa1-6 cells (Supplementary Figure S1A,B) and C57BL/6 mice (Supplementary Figure S2A,B). We then investigated whether p53 participates in SIRT6-mediated inhibition of NAFLD. Hepatic overexpression of human SIRT6 reduced the ratio of liver to body weight and hepatic TG and FFA levels in Western diet-fed C57BL/6 mice, which were blunted when p53 was overexpressed in the liver (Supplementary Figure S2C–F). Overexpression of human SIRT6 inhibited hepatic apoptosis in both control mice and p53-overpressing mice. p53 overexpression also normalized hepatic apoptosis in SIRT6-overexpressing mice (Supplementary Figure S2G–H). Thus, the data in Supplementary Figure S2 suggest that p53 plays a role in hepatic SIRT6-mediated inhibition of NAFLD. 

### 3.5. Hepatic SIRT6 can Sufficiently Protect against the Development of Atherosclerosis in Ldlr^−/−^ Mice

**Figure 4:**
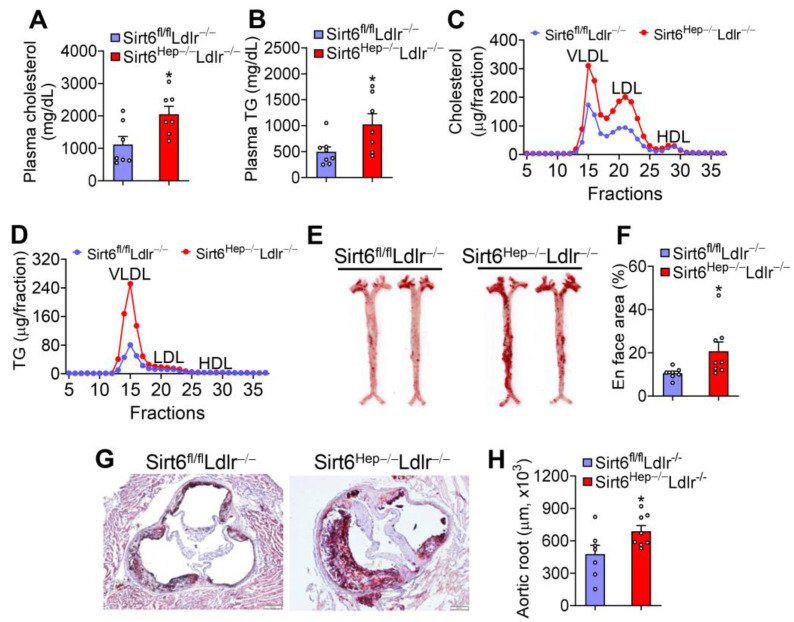
**Loss of hepatic SIRT6 aggravates the development of atherosclerosis in *Ldlr*^−/−^ mice.** *Sirt6^fl/fl^Ldlr*^−/−^ mice and *Sirt6^Hep^*^−/−^*Ldlr*^−/−^ mice were fed a Western diet for 16 weeks (n = 8 per group). Plasma total cholesterol (**A**) and triglyceride (**B**) levels were quantified, and FPLC analysis of plasma cholesterol (**C**) or triglyceride (**D**) lipoprotein profiles were determined. *En face* aorta lesions were stained with Oil Red O (**E**) and quantified (**F**). The lesions of aortic roots were stained (**G**) and quantified (**H**). All data are expressed as mean ± SEM. Data points in the graphs represent an individual mouse or a biological measurement. Statistical analysis was performed using a student *t*-test. * *p* < 0.05.

The role of hepatic SIRT6 in atherosclerosis has not been investigated to date. Loss of hepatocyte *Sirt6* in *Ldlr*^−/−^ mice raised plasma total cholesterol by 41% (Figure 4A) and plasma triglyceride levels by 206% (Figure 4B). Analysis of plasma lipoprotein profiles by fast protein liquid chromatography (FPLC) showed that *Sirt6^Hep^*^−/−^*Ldlr*^−/−^ mice had higher levels of VLDL-C, LDL-C (Figure 4C), and VLDL-TG (Figure 4D). Consistent with changes in plasma lipid levels, *Sirt6^Hep^*^−/−^*Ldlr*^−/−^ mice had a 200% and 144% increase in en face lesions (Figure 4E,F) and aortic root lesions (Figure 4G,H), respectively. 

**Figure 5:**
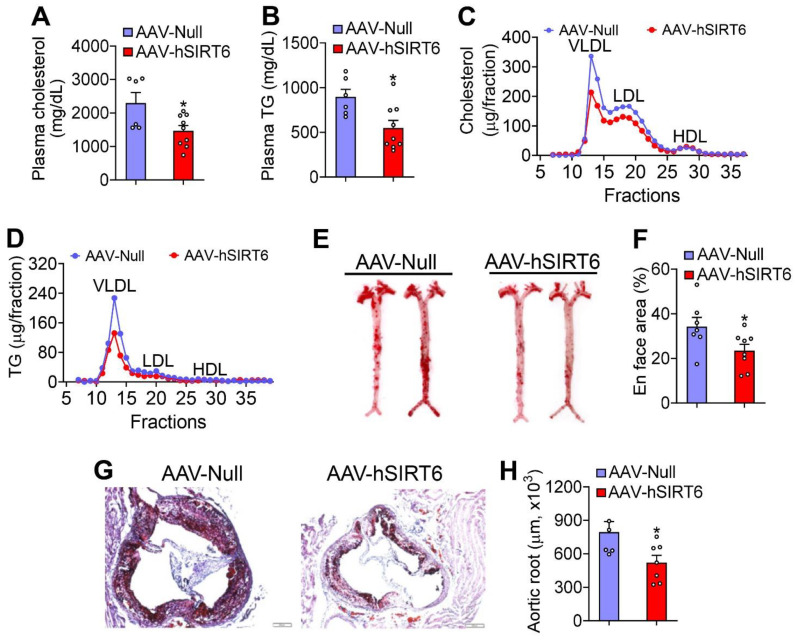
**Over-expression of hepatic SIRT6 attenuates the development of atherosclerosis in *Ldlr*^−/−^ mice.** *Ldlr*^−/−^ mice were i.v. injected with AAV8-ALB-Null or AAV8-ALB-hSIRT6 and fed a Western diet for 16 weeks (n = 8 per group). Plasma total cholesterol (**A**) and triglyceride (**B**) levels were quantified. FPLC analysis of plasma cholesterol (**C**) or triglyceride (**D**) lipoprotein profiles was determined. En face aorta lesions were stained with Oil Red O (**E**) and quantified (**F**). The lesions on the aortic roots were stained (**G**) and quantified (**H**). All data are expressed as mean ± SEM. Data points in the graphs represent an individual mouse or a biological measurement. Statistical analysis was performed using a student *t*-test. * *p* < 0.05.

By contrast, total cholesterol (Figure 5A) and triglyceride (Figure 5B) levels in plasma from *Ldlr*^−/−^ mice overexpressing hepatic SIRT6 were reduced by 37% and 39%, respectively. FPLC analysis showed that SIRT6 overexpression reduced VLDL-C, LDL-C (Figure 5C), and VLDL-TG (Figure 5D). As a result, overexpression of hepatic SIRT6 reduced the lesion size of en face aortas (Figure 5E,F) and aortic roots (Figure 5G,H) by 32% and 35%, respectively. In summary, the data in Figure 4 and Figure 5 demonstrate that hepatic SIRT6 sufficiently protects against diet-induced atherosclerosis.

### 3.6. Hepatic SIRT6 is Critical for Regulating Intestinal Cholesterol and Fat Absorption and VLDL Secretion

**Figure 6:**
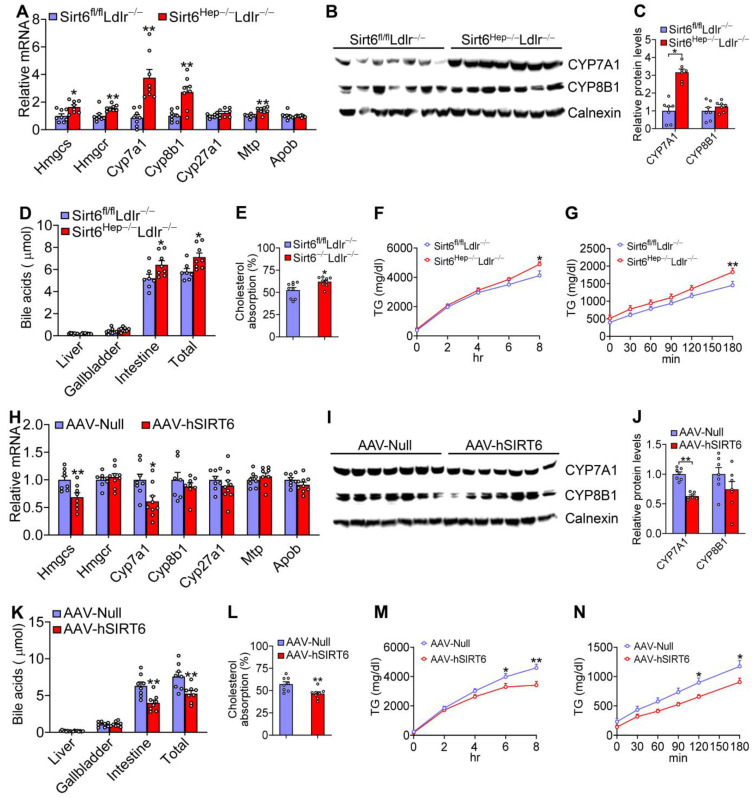
**Hepatic SIRT6 inhibits intestinal cholesterol and fat absorption and VLDL secretion.** (**A**–**G**) *Sirt6^fl/fl^Ldlr*^−/−^ mice and *Sirt6^Hep^*^−/−^*Ldlr*^−/−^ mice were fed a Western diet for 16 weeks (n = 8 per group). Hepatic mRNA (**A**) and protein (**B**,**C**) levels were quantified. Bile acid pool size (**D**), intestinal cholesterol (**E**) and fat (**F**) absorption, and hepatic VLDL secretion (**G**) were determined. (**H**–**N**) *Ldlr*^−/−^ mice were i.v. injected with AAV8-ALB-Null or AAV8-ALB-hSIRT6 and fed a Western diet for 16 weeks (n = 8 per group). Hepatic mRNA (**H**) and protein (**I**,**J**) levels were quantified. Bile acid pool size (**K**), intestinal cholesterol (**L**) and fat (**M**) absorption, and hepatic VLDL secretion (**N**) were determined. All data are expressed as mean ± SEM. Data points in the graphs represent an individual mouse or a biological measurement. Statistical analysis was performed using a student *t*-test (**A**,**C**–**E**,**H**,**J**–**L**) or two-way ANOVA (**F**,**G**,**M**,**N**). * *p* < 0.05, ** *p* < 0.01.

The inhibited development of atherosclerosis by SIRT6 in *Ldlr*^−/−^ mice suggests that LDLR does participate in SIRT6-mediated suppression of atherosclerosis. In *Ldlr*^−/−^ mice, loss of hepatocyte *Sirt6* reduced hepatic mRNA levels of genes involved in cholesterol synthesis (HMG-CoA synthase (*Hmgcs*), HMG-CoA reductase (*Hmgcr*)), bile acid synthesis (cholesterol 7α-hydroxylase (*Cyp7a1*), sterol 12α-hydroxylase (*Cyp8b1*)), and VLDL secretion (microsomal triglyceride transfer protein (*Mtp*)) (Figure 6A). There were only slight changes in *Cyp27a1* or *Apob* expression (Figure 6A). *Sirt6* ablation raised hepatic CYP7A1 protein levels by ~3.1-fold (Figure 6B,C) while also increasing intestinal bile acid levels and the bile acid pool size (Figure 6D). Consistent with the changes in gene expression and bile acid levels, *Sirt6^Hep^*^−/−^*Ldlr*^−/−^ mice saw an increase in cholesterol (Figure 6E) and fat (Figure 6F) absorption from the intestine and VLDL secretion from the liver (Figure 6G). 

Hepatic *Cyp7a1* mRNA, protein, and *Hmgcs* mRNA levels were reduced in *Ldlr*^−/−^ mice overexpressing hepatic SIRT6 (Figure 6H–J). Interestingly, there were only minor changes in *Hmgcr*, *Cyp8b1*, *Cyp27a1*, *Mtp,* or *Apob* expression (Figure 6H–J). Consistent with the inhibition of CYP7A1 expression, hepatic SIRT6 overexpression reduced bile acid pool size (Figure 6K), cholesterol and fat absorption from the intestine (Figure 6L,M), and VLDL secretion from the liver (Figure 6N). 

In summary, the data in Figure 6 suggest that hepatic SIRT6 lowers plasma lipid levels by inhibiting intestinal cholesterol and fat absorption and hepatic VLDL secretion. 

### 3.7. Hepatic SIRT6 Is Required for Preventing Western Diet-Induced Obesity

**Figure 7:**
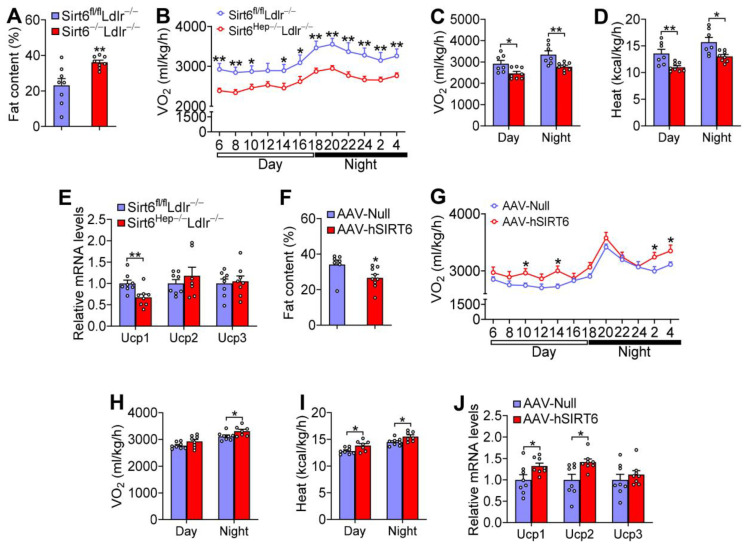
**Hepatic SIRT6 protects against Western diet-induced obesity by inducing thermogenesis in *Ldlr*^−/−^ mice**. (**A**–**E**) *Sirt6^fl/fl^Ldlr*^−/−^ mice and *Sirt6^Hep^*^−/−^*Ldlr*^−/−^ mice were fed a Western diet for 16 weeks (n = 8 per group). Fat content (**A**), oxygen consumption (**B**,**C**), heat production (**D**), and mRNA levels in brown adipose tissue (BAT) (**E**) were determined. (**F**–**J**) *Ldlr*^−/−^ mice were i.v. injected with AAV8-ALB-Null or AAV8-ALB-hSIRT6 and fed a Western diet for 16 weeks (n = 8 per group). Fat content (**F**), oxygen consumption (**G**,**H**), heat production (**I**), and mRNA levels in BAT (**J**) were determined. All data are expressed as mean ± SEM. Data points in the graphs represent an individual mouse or a biological measurement. Statistical analysis was performed using a student *t*-test (**A**,**E**,**F**,**J**) or two-way ANOVA (**B**–**D**,**G**–**I**). * *p* < 0.05, ** *p* < 0.01.

Obesity is a major risk factor for metabolic disorders. Loss of hepatocyte *Sirt6* in *Ldlr*^−/−^ mice did not affect food intake (Supplementary Figure S3A), but increased body fat content by 156% (Figure 7A). CLAMS studies showed that *Sirt6^Hep^*^−/−^*Ldlr*^−/−^ mice had reduced oxygen consumption (Figure 7B,C) and heat production (Figure 7D) during the day and night, whereas the respiratory exchange ratio (RER) was unchanged (Supplementary Figure S3B). At gene expression levels, loss of hepatocyte *Sirt6* reduced uncoupled protein 1 (*Ucp1*) expression in brown adipose tissue (BAT) (Figure 7E). 

Hepatic SIRT6 overexpression decreased fat content by 22% (Figure 7F), increased oxygen consumption (Figure 7G,H) and heat production (Figure 7I) as well as *Ucp1* and *Ucp2* expression in BAT (Figure 7J). By contrast, there was no change in food intake or RER (Supplementary Figure S3C,D). Thus, hepatic SIRT6 is required to prevent diet-induced obesity by regulating energy expenditure.

## 4. Discussion

The role of hepatic SIRT6 in atherosclerosis or obesity has not been investigated before. In addition, the role of hepatic SIRT6 in the development of NAFLD has not been fully understood. In this work, we show that loss of hepatocyte SIRT6 aggravates Western diet-induced NAFLD, atherosclerosis, and obesity in *Ldlr*^−/−^ mice. By contrast, AAV-mediated overexpression of human SIRT6 in the liver has opposite effects. Mechanistically, our data suggest that hepatocyte SIRT6 likely inhibits the development of NAFLD by suppressing de novo lipogenesis, lipid droplet formation, the p53 pathway, and inflammation. It also prevents the development of atherosclerosis by inhibiting intestinal fat and cholesterol absorption and hepatic VLDL secretion. 

SIRT6 has been shown to lower plasma lipid levels by an unknown mechanism [25]. SIRT6 reportedly reduces LDL-C levels by inhibiting PCSK9-mediated LDLR degradation receptor degradation [6]. However, the loss or overexpression of hepatic SIRT6 markedly regulates plasma LDL-C levels and atherogenesis in *Ldlr*^−/−^ mice, suggesting that LDLR does not mediate SIRT6’s effects on plasma LDL-C levels. Our data show that hepatic SIRT6 inhibits cholesterol and fat absorption from the intestine and VLDL secretion from the liver. Furthermore, SIRT6 reduces CYP7A1 expression and bile acid pool size, which may contribute to changes in intestinal lipid absorption and hepatic VLDL secretion, since *Cyp7a1*^−/−^ mice displayed reduced cholesterol absorption [26] and over-expression of hepatic CYP7A1 increased VLDL secretion [27].

Our data clearly show that hepatocyte SIRT6 inhibits the development of NAFL and NASH. Previous studies showed that hepatic SIRT6 inhibits de novo lipogenesis (DNL) by suppressing LXR, ChREBP, and SREBP1 [5], and inducing FAO via PPARα [3]. Our data suggest that hepatocyte SIRT6 likely inhibits NAFL by inhibiting DNL and lipid droplet formation. The role of hepatocyte SIRT6 in NASH development has not been well understood. SIRT6 in stellate cells inhibits HSC activation via the deacetylation of Smad2 [8], Smad3 [9], TGFβ [10], YAP, and TAZ [11]. Hepatocyte-specific *Sirt6* deletion reportedly leads to NASH development by upregulating Bach1, an Nrf2 repressor [12]. Lipotoxicity and apoptosis play a key role in the pathogenesis of NASH [22,28,29,30]. p53 is also known to promote apoptosis [21] and NASH development [22,23]. Our data show that SIRT6 reduces hepatic FFA, FC and p53 levels and apoptosis, partially explaining how hepatocyte SIRT6 inhibits NASH development. 

We also found that hepatic SIRT6 inhibits obesity. Although SIRT6 has been shown to regulate obesity [31], it has not been investigated whether hepatic SIRT6 regulates obesity. Our data show that hepatic SIRT6 reduces obesity by inducing UCP1 in BAT and energy expenditure. However, the precise mechanism remains elusive. *Cyp7a1*^−/−^ mice are resistant to diet-induced obesity via a yet-to-be-determined mechanism [27]. Our data show that SIRT6 inhibits CYP7A1 expression. Thus, hepatic SIRT6 likely inhibits diet-induced obesity by suppressing hepatic CYP7A1. 

In summary, we identified hepatic SIRT6 as a key regulator of NAFLD, atherosclerosis, and obesity. Targeting hepatocyte SIRT6 may be useful for treating common metabolic disorders. One limitation of the current study is that we did not investigate atherosclerotic plaque composition, which will be further characterized in future work.

## Data Availability

The data are available from Y. Zhang upon request.

## Supplementary Materials

The following supporting information can be downloaded at: https://www.mdpi.com/article/10.3390/cells12152009/s1, Table S1: qPCR primers; Figure S1: SIRT6 reduces P53 protein levels in Hepa1-6 cells; Figure S2: SIRT6 reduces hepatic apoptosis and lipid levels partly via P53; Figure S3: Hepatic SIRT6 does not affect food intake or RER in Western diet-fed *Ldlr*^−/−^ mice.
