# Supplementary material for: Hepatocyte Sirtuin 6 Protects against Atherosclerosis and Steatohepatitis by Regulating Lipid Homeostasis
Source: Cells. 2023 Aug 5;12(15):2009. doi: 10.3390/cells12152009 (PMC10417046; doi:10.3390/cells12152009)
Supplement: Supplementary file 1 [file cells-12-02009-s001.zip › cells-2475574-SI.pdf]

Hepatocyte Sirtuin 6 protects against atherosclerosis and steatohepatitis by regulating lipid homeostasis

Yingdong Zhu, Shuwei Hu, Xiaoli Pan, Raja Gopoju, Fathima N. Cassim Bawa, Liya Yin, Yanyong Xu, and Yanqiao Zhang

	Table 1. qPCR primers	
**Primer name**	**Forward (5' to 3')**	**Reverse (5' to 3')**
Acc1	AGTGGAGCTAGAATTGGACTTG	ACAGTGGACAGAATTGAGGG
Acc2	AGTCTTCCGTGCCTTTGTAC	TTCTGCAAACTCATCCCTCG
Apob	CGTGGGCTCCAGCATTCTA	TCACCAGTCATTTCTGCCTTTG
Ccl2	GTCCCTGTCATGCTTCTGG	GCTCTCCAGCCTACTCATTG
Cd36	TGACTGGGAAAATCAAGCTCC	CCAGTGTATATGTAGGCTCATCC
Cidea	GAATAGCCAGAGTCACCTTCG	AGCAGATTCCTTAACACGGC
Cideb	TCCGTGTCTGTGATCATAAGC	GTTAGCACTCCACGTAGCAG
Col1a1	CATAAAGGGTCATCGTGGCT	TTGAGTCCGTCTTTGCCAG
Col3a1	GAAGTCTCTGAAGCTGATGGG	TTGCCTTGCGTGTTTGATATTC
Cyp7a1	CACCATTCCTGCAACCTTCTGG	ATGGCATTCCCTCCAGAGCTGA
Cyp8b1	GTTTCTGGGTCCTCTTATTCCTG	TGGGAGTGAAAGTGAACGAC
Cyp27a1	GCCTCACCTATGGGATCTTCA	TCAAAGCCTGACGCAGATG
F4/80	ACCACAATACCTACATGCACC	AAGCAGGCGAGGAAAAGATAG
Fasn	GCTGCGGAAACTTCAGGAAAT	AGAGACGTGTCACTCCTGGACTT
Fsp27a	GCCACGCGGTATTGCCAGGA	GGGTCTCCCGGCTGGGCTTA
Fsp27b	GTGACCACAGCTTGGGTCGGA	GGGTCTCCCGGCTGGGCTTA
Hmgcr	CTTGTGGAATGCCTTGTGATTG	AGCCGAAGCAGCACATGAT
Hmgcs	GCCGTGAACTGGGTCGAA	GCATATATAGCAATGTCTCCTGCAA
Il1b	CCTGAACTCAACTGTGAAATGC	GCGAGATTTGAAGCTGGATG
Mtp	TCCTCTATGCCTGTGGCTTT	TCTCTGATGTCGTTGCTTGC
Plin2	GGATAAGCTCTATGTCTCGTGG	GTCTGGCATGTAGTCTGGAG
Plin3	ACCTGAGGACTTTGCAACTG	CGTGGAACTGATAAGAGGCAG
Plin4	AAACAGCAACAGACCCCTC	AACTTCCCATGTCCTTGTCTC
Plin5	CATGACTGAGGCTGAGCTAG	GAGTGTTCATAGGCGAGATGG
Scd1	CATTCAATCCCGGGAGAATA	CTGGCAGAGTAGTCGAAGGG
Tgfb	CCTGAGTGGCTGTCTTTTGA	CGTGGAGTTTGTTATCTTTGCTG
Timp1	CTCAAAGACCTATAGTGCTGGC	CAAAGTGACGGCTCTGGTAG
Tnfa	CCCTCCAGAAAAGACACCATG	GCCACAAGCAGGAATGAGAAG

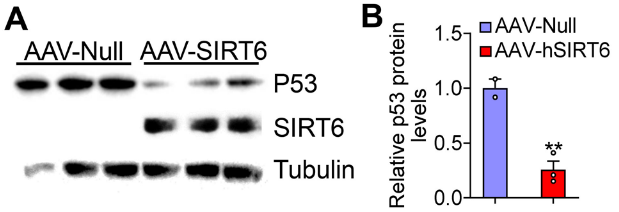

Supplementary Figure 1. SIRT6 reduces P53 protein levels in Hepa1-6 cells
Hepa1-6 cells were transfected with AAV8-ALB-Null or AAV8-ALB-hSIRT6 plasmid DNA. After 30 h, Western blot assays were performed (A) and P53 protein levels were quantified (B). All data are expressed as mean±SEM. ***P*<0.01

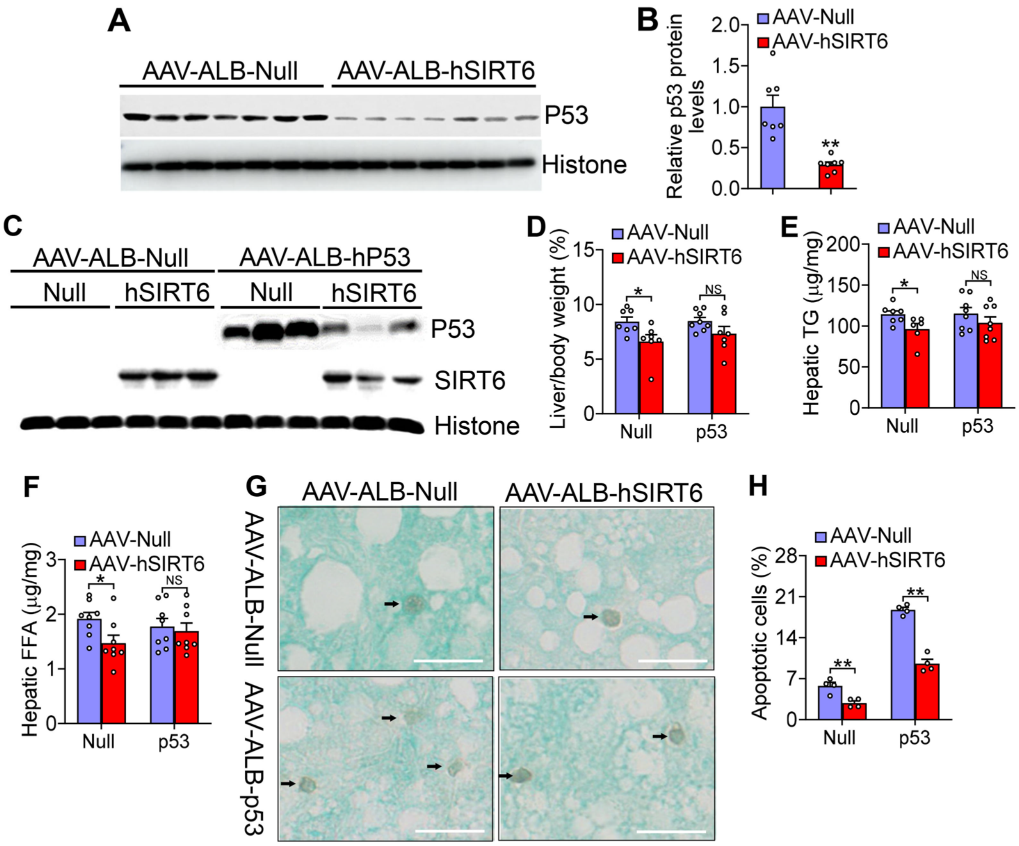

Supplementary Figure 2. SIRT6 reduces hepatic apoptosis and lipid levels partly via P53

(A and B) Hepatic protein levels in C57BL/6 mice injected with AAV8-ALB-Null or AAV8-ALB-hSIRT6 (n=7 per group). (C-H) C57BL/6 mice were i.v. injected with AAV8-ALB-Null, AAV8-ALB-hSIRT6 and/or AAV8-ALB-hP53 (n=7-8), and then fed a Western diet for 16 weeks. Western blot assays were performed (C) and the ratio of liver to body weight (%) (D) as well as hepatic triglyceride (TG) (E) and free fatty acid (FFA) (F) levels were measured. TUNEL staining was performed (G) and apoptotic cells (%) were calculated (H). In (G), arrows point to apoptotic cells. All data are expressed as mean±SEM. Statistical analysis was performed using a student *t*-test (B) or two-way ANOVA (D-F, H). **P*<0.05, ***P*<0.01

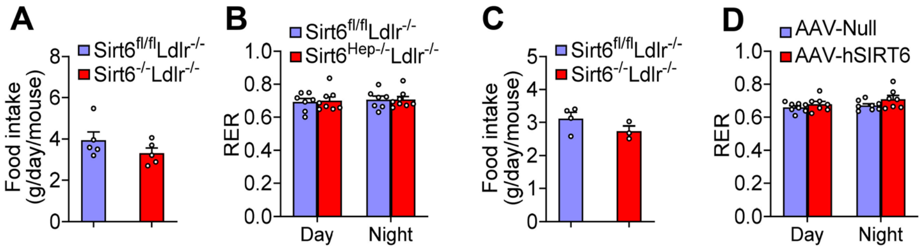

Supplementary Figure. 3. Hepatic SIRT6 has no effect on food intake or RER in Western diet-fed Ldlr$^{-/-}$ mice

(A-B) Sirt6$^{fl/fl}$Ldlr$^{-/-}$ mice and Sirt6$^{Hep-/-}$Ldlr$^{-/-}$ mice were fed a Western diet for 16 weeks (n=8 per group). Food intake (A) and respiration exchange ratio (RER) (B) were determined. (C-D) Ldlr$^{-/-}$ mice were i.v. injected with AAV8-ALB-Null or AAV8-ALB-hSIRT6 and then fed a Western diet for 16 weeks (n=8 per group). Food intake (C) and RER (D) were determined. All data are expressed as mean±SEM
